# Access to Complex Abortion Care Service and Planning Improved through a Toll-Free Telephone Resource Line

**DOI:** 10.1155/2014/913241

**Published:** 2014-02-13

**Authors:** Wendy V. Norman, Barbara Hestrin, Royce Dueck

**Affiliations:** ^1^Department of Family Practice, Faculty of Medicine, University of British Columbia, Vancouver, BC, Canada V6T 1Z3; ^2^Contraception Access Research Team, Women's Health Research Institute, BC Women's Hospital and Health Centre, Vancouver, BC, Canada V6H 3N1; ^3^BC Women's Hospital and Health Centre, Vancouver, BC, Canada V6H 3N1; ^4^Pregnancy Options Service, BC Women's Hospital and Health Centre, Vancouver, BC, Canada V6H 3N1

## Abstract

*Background*. Providing equitable access to the full range of reproductive health services over wide geographic areas presents significant challenges to any health system. We present a review of a service provision model which has provided improved access to abortion care; support for complex issues experienced by women seeking nonjudgmental family planning health services; and a mechanism to collect information on access barriers. The toll-free pregnancy options service (POS) of British Columbia Women's Hospital and Health Centre sought to improve access to services and overcome barriers experienced by women seeking abortion. *Methods*. We describe the development and implementation of a province-wide toll-free telephone counseling and access facilitation service, including establishment of a provincial network of local abortion service providers in the Canadian province of British Columbia from 1998 to 2010. *Results*. Over 2000 women annually access service via the POS line, networks of care providers are established and linked to central support, and central program planners receive timely information on new service gaps and access barriers. *Conclusion*. This novel service has been successful in addressing inequities and access barriers identified as priorities before service establishment. The service provided unanticipated benefits to health care planning and monitoring of provincial health care related service delivery and gaps. This model for low cost health service delivery may realize similar benefits when applied to other health care systems where access and referral barriers exist.

## 1. Background

The pregnancy options service (POS) is toll-free telephone service in the Canadian province of British Columbia (BC), established to improve rural and remote access to counseling and referral for induced abortion services.

We review the initial decade of this service to assist those facing access and referral barriers and highlight the value of a low cost model which identifies health care gaps and informs health service planning, while improving access to the full range of reproductive options and counseling, for women throughout a large geographic area.

### 1.1. Context and History

Induced abortion is a common procedure in Canada currently experienced by a third of Canadian women [1, 2]. Abortion services in BC are nearly exclusively located in urban areas in the extreme southwest. BC, having the area of Germany and France together although only a population of approximately four million, presents significant access barriers to abortion service for those in rural and remote areas [3]. Barriers to accessing care disproportionally affect those with lower incomes or in rural and remote areas [4, 5].

In 1997 British Columbia Women's Hospital and Health Centre (BC Women's), the tertiary maternity and women's health center for the province, implemented POS within their abortion service, in response to government recommendations to address the service distribution gap.

## 2. Methods

The POS program had three goals related to unintended and abnormal pregnancies: improve access to care by establishing a provincial counseling, information, and referral service; provide access to counseling for women; and support local health regions to identify and meet service gaps. Implementation was planned in phases. *Phase I*: establish a province-wide network of supportive professionals providing counseling and/or abortion services. *Phase II*: design and implement a province-wide toll-free telephone counseling/information/referral service (POS); and a functional, secure data base. *Phase III*: analyze information from Phase I and Phase II to develop and implement strategies for improving abortion access.

### 2.1. Implementation

*Phase I.* To identify care providers, while respecting personal safety and confidentiality, a letter from BC Women's, distributed to all provincial general practitioners and obstetrician-gynecologists, and one to counselors, introduced the service and invited participation in the network as a service provider or as a supportive referring physician. Letters were followed up by community meetings. Physicians and counselors from every health region responded positively to these invitations and participated in the meetings and subsequent planning process and network establishment. Both networks were established over an eight-month period and involved eleven community consultations. The initial database included participants in approximately 50 communities representing nearly every health service delivery area in the province. A list of supportive physicians and community health workers within regions where no services were available was also developed. A secure purposed-designed database accessible only to POS staff and not linked in any way to other networks was developed. As professionals associated with the provision of abortion service in Canada and the United States have been targeted by terrorist tactics (shooting and bombing), the security of the identities of the members of the network was considered of the highest importance. 

*Phase II.* Implementation of the toll-free telephone service began in March 1998.

*Phase III.* Health service planning was guided by needs delineated within the community consultations, the development of a database detailing health professional services and locations, and ongoing identification of health care gaps. POS staff assisted callers to overcome barriers relating to family, financial, and citizenship issues. The POS initial purpose had included “to support local health regions to identify and meet service gaps.” However, it had not been anticipated that the tertiary care abortion service would also benefit from identification of service gaps. Their real-time awareness of access barriers, previously unidentified by the health system, unexpectedly offered an enhanced ability to address health system gaps in access, distribution, and delivery of health services.

## 3. Results

### 3.1. Ten Years of Operation

Data on access barriers collected over ten years have informed provincial initiatives and helped develop several strategies to improve access and availability of services.

- The provision of support for abortion providers following targeted violent attacks on abortion providers in Canada.
- *The Abortion and Quality Assurance Project* (2002) was an initiative undertaken by the Ministry of Health of the Government of BC, specifically in response to variations in quality and access to services detected through the POS. This government health service improvement initiative examined policies related to access, quality of care, and knowledge gaps that could be addressed with future services.
- A BC survey regarding access to contraception, counseling, and abortion services (*Access to Abortion Survey Report, 2003*).
- Two regional centers and two smaller communities were assisted to establish new abortion services.

**Figure 1:**
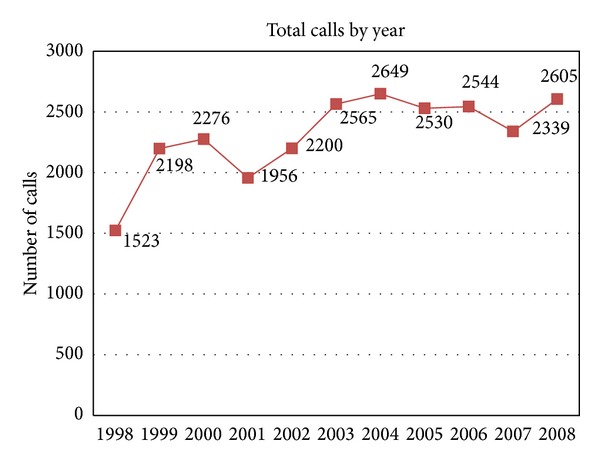
Total calls to the toll-free line, by year 1998–2008. The total number of calls received by the toll-free service increased gradually over the first five years then remained relatively stable at about 2500 (+/− 100) calls per year.

POS answered over 2000 calls per year providing direct access to a capable and responsive staff person with the ability to mobilize or utilize appropriate resources and information.  Figure 1 details the total number of calls received by the toll-free service and the rate at which these increased gradually over the first five years then remained relatively stable at about 2500 (+/− 100) calls per year over the next five years. At the same time the service provided an unanticipated sentinel function. Between 1998 and 2010 POS noted a 62% decline in the number of physicians providing abortion service outside the main population center, spurring research to determine the etiology and any addressable barriers contributing to this decline [6, 7]. The failure of medical schools and residency training programs [8, 9] to include sufficient study of abortion care may contribute to this health system gap. POS data have provided impetus for BC Women's to increase training in abortion. Another situation illustrating the POS's sentinel function occurred in 2007 when the United States immigration regulations changed to require a valid passport for entry [10]. Until this time many who required abortion services for pregnancies beyond 20-week gestation had received care at American clinics. Women in this situation frequently include those living with substance use and mental health issues, experiencing interpersonal violence and poverty, and having lower education levels [11–13]. The POS immediately detected that a significant portion of women requiring this service did not have a passport; the time sensitive nature for this procedure would not allow sufficient time to acquire one. POS colocation within BC's tertiary abortion facility allowed timely discussions with health service administrators and rapid initiation of appropriate services in BC.

**Figure 2:**
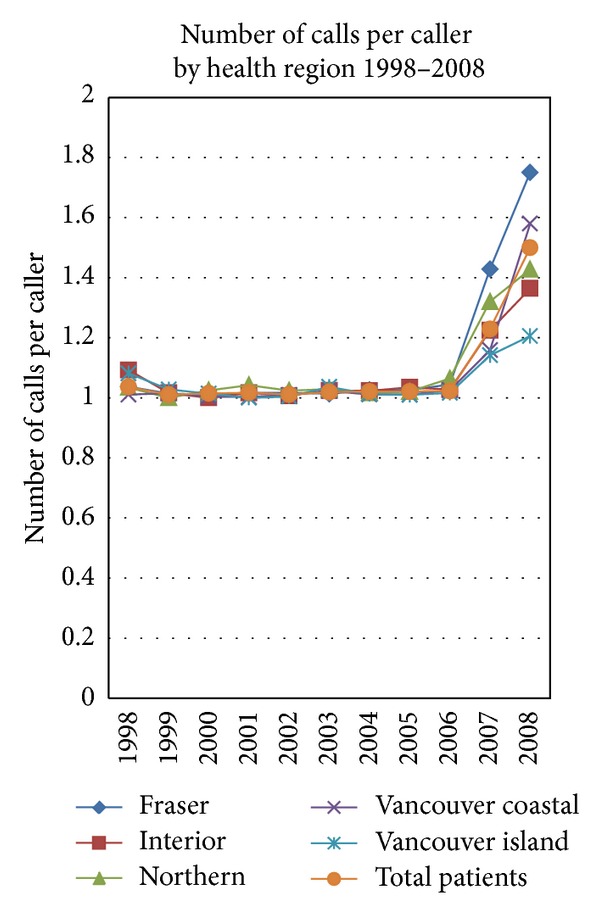
Number of calls per caller by health region, 1998–2008. The POS service initially provided chiefly information and referral consistent with a single call per caller, serving both health professionals and women seeking care. From 2006 to 2008 there was an increasing trend toward “case management” where the complex needs of women seeking options for a second trimester pregnancy were handled by the service utilizing a series of calls with each individual.

Figure 2 illustrates the qualitative alteration in service delivery over time. The POS service initially provided chiefly information and referral consistent with a single call per caller, serving both health professionals and women seeking care. From 2006 to 2008 there was an increasing trend toward “case management” where the complex needs of women seeking options for a second trimester pregnancy were handled by the service utilizing a series of calls with each individual. The increase seen in the number of calls per caller to the POS line is due to a number of factors. The majority of women with unintended pregnancies require only a single call for information and referral. Gradually after service initiation, health professionals in remote regions began to utilize the POS decision-management pathways to improve local case management. For example, the management of a woman requesting an abortion has a different sequence of diagnostic testing (laboratory or ultrasound requirements) and a different sequence, timing, or location for services (e.g., medication abortion compared to a first trimester compared to a multiday second trimester surgical abortion). Over time POS has increasingly evolved into a complex case management service.

**Figure 3:**
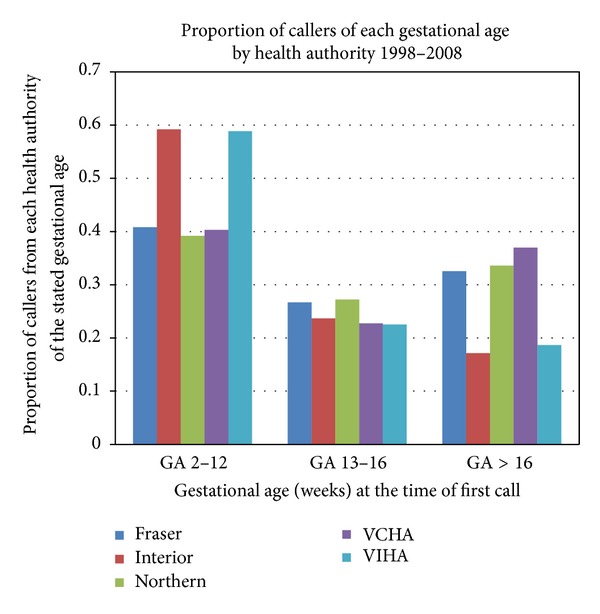
Proportion of callers of each gestational age by health authority, among those for whom the gestational age is known (*n* = 7092). Although in Canada pregnancies beyond 16-week gestational age at the time of termination are fewer than 5% of all induced abortions, they represent a significant proportion of the users of the pregnancy options toll-free line.

Women with unintended pregnancy of advanced gestation, and those with an abnormal pregnancy, are a small proportion of those seeking abortion services, but a significant proportion of those seeking service with the POS line (Figure 3). In Canada more than 90 percent of abortions occur before 12 weeks and fewer than 3% beyond 16 weeks [4, 14] as compared to the proportion of callers to POS with nearly 30% beyond 16-week gestation. The POS expertise in achieving access for women led to a high level of trust among local health system administrators and local health service providers. Over time, POS increasingly played a role as a key informant in relevant aspects of both central and regional health care plannings. 

*Next Steps.* A formal stakeholder evaluation of POS is underway and iterative refinement of the POS data collection system and resources list continues.

## 4. Conclusion

The POS provides health system administrators with a service provision model to identify and address barriers to access health services over wide geographic areas. The toll-free POS counseling and referral line has provided direct assistance and support for marginalized and vulnerable women. The greatest value may be to health system administrators through provision of real-time information on health service needs and gaps, thus informing continuous integrated improvements to health care planning. The benefit of continuously monitoring health service needs, while providing direct assistance to the most vulnerable in need of services irrespective of geographic barriers, may be applicable in other areas of health care where access barriers exist.
